# Effect of almond consumption on insulin sensitivity and serum lipids among Asian Indian adults with overweight and obesity– A randomized controlled trial

**DOI:** 10.3389/fnut.2022.1055923

**Published:** 2023-01-10

**Authors:** Rajagopal Gayathri, Kuzhandhaivelu Abirami, Natarajan Kalpana, Valangaiman Sriram Manasa, Vasudevan Sudha, Shanmugam Shobana, Raman Ganesh Jeevan, Vasudevan Kavitha, Karthikeyan Parkavi, Ranjit Mohan Anjana, Ranjit Unnikrishnan, Kuppan Gokulakrishnan, D. Annette Beatrice, Kamala Krishnaswamy, Rajendra Pradeepa, Richard D. Mattes, Jordi Salas-Salvadó, Walter Willett, Viswanathan Mohan

**Affiliations:** ^1^Department of Foods Nutrition and Dietetics Research, Madras Diabetes Research Foundation, Chennai, Tamil Nadu, India; ^2^Department of Biochemistry, University of Madras, Chennai, Tamil Nadu, India; ^3^Department of Diabetes Food Technology, Madras Diabetes Research Foundation, Chennai, Tamil Nadu, India; ^4^Department of Diabetology, Madras Diabetes Research Foundation, Chennai, Tamil Nadu, India; ^5^Department of Neurochemistry, National Institute of Mental Health and Neuro Sciences (NIMHANS), Bengaluru, Karnataka, India; ^6^Department of Home Science, Women’s Christian College, Chennai, Tamil Nadu, India; ^7^Department of Research Operations, Madras Diabetes Research Foundation, Chennai, Tamil Nadu, India; ^8^Department of Nutrition Science, College of Health and Human Sciences, Purdue University, West Lafayette, IN, United States; ^9^Human Nutrition Unit, Department of Biochemistry and Biotechnology, Institut d’Investigació Sanitària Pere Virgili, Universitat Rovira i Virgili, Reus, Spain; ^10^Consorcio CIBER, M.P. Fisiopatología de la Obesidad y Nutrición (CIBERObn), Instituto de Salud Carlos III (ISCIII), Madrid, Spain; ^11^Department of Nutrition, Harvard School of Public Health, Boston, MA, United States; ^12^Department of Epidemiology, Harvard School of Public Health, Boston, MA, United States

**Keywords:** almond, overweight and obesity, insulin resistance, serum lipid, insulin sensitivity, Asian–Indian

## Abstract

**Background:**

Asian Indians have an increased susceptibility to type 2 diabetes and premature coronary artery disease. Nuts, like almonds, are rich in unsaturated fat and micronutrients with known health benefits.

**Objectives:**

This study aimed to assess the efficacy of almonds for reduction of insulin resistance and improving lipid profile in overweight Asian Indian adults.

**Methods:**

This parallel-arm, randomized, controlled trial was conducted in Chennai, India on 400 participants aged 25–65 years with a body mass index ≥ 23 kg/m^2^. The intervention group received 43 g of almonds/day for 12 weeks, while the control group was advised to consume a customary diet but to avoid nuts. Anthropometric, clinical, and dietary data were assessed at periodic intervals. Glucose tolerance, serum insulin, glycated hemoglobin, C-peptide and lipid profile were assessed at baseline and end of the study. Insulin resistance (homeostasis assessment model-HOMA IR) and oral insulin disposition index (DIo) were calculated.

**Results:**

A total of 352 participants completed the study. Significant improvement was seen in DIo [mean (95% CI) = + 0.7 mmol/L (0.1, 1.3); *p* = 0.03], HOMA IR (−0.4 (−0.7, −0.04; *p* = 0.03) and total cholesterol (−5.4 mg/dl (−10.2, −0.6); *p* = 0.03) in the intervention group compared to the control group. Incremental area under the curve (IAUC) and mean amplitude of glycemic excursion (MAGE) assessed using continuous glucose monitoring systems were also significantly lower in the intervention group. Dietary 24-h recalls showed a higher significant reduction in carbohydrate and increase in mono unsaturated fatty acid (MUFA) and polyunsaturated fatty acids (PUFA) intake in the intervention group compared to the control group.

**Conclusion:**

Daily consumption of almonds increased the intake of MUFA with decrease in carbohydrate calories and decreases insulin resistance, improves insulin sensitivity and lowers serum cholesterol in Asian Indians with overweight/obesity. These effects in the long run could aid in reducing the risk of diabetes and other cardiometabolic disease.

## Introduction

Obesity has become a global epidemic and a major contributor to non-communicable diseases (NCDs) including diabetes and cardiovascular disease (CVD). Worldwide prevalence of overweight and obesity is estimated to be 39 and 13%, respectively (1). A national study reported the prevalence of both generalized (21%) and abdominal obesity (25%) to be high among Indian adults (2). India is home to over 135 million individuals with obesity (3) and obesity rates are expected to triple by 2040 in India (4). Intake of energy dense foods with low nutritional value and increased sedentary behavior due to societal and environmental changes have been identified as key risk factors for obesity and related NCDs such as type 2 diabetes (T2D) which now tend to occur at a much earlier ages than previously in this population (5). Unhealthy diets are also an important contributor to mortality due to NCDs (5). Asian Indians consume diets high in refined carbohydrate staples like polished white rice, refined wheat and added sugars with low intake of fruits and vegetables (6, 7). The diets also tend to be low in monounsaturated fatty acid (MUFA) and dietary fiber. These dietary practices combined with low physical activity levels increase the risk of obesity, insulin resistance, T2D, and CVD (8, 9). The Global Burden of Diseases study identified 10 prominent dietary risk factors of which low intake of fruits, nuts, and seeds significantly contributed to disability adjusted life years (DALYs) (10). Thus, it is reasonable to hypothesize that providing healthier foods could improve the cardio-metabolic health of individuals especially in the south Asian context (11).

Insulin resistance and pancreatic β cell dysfunction are the two most important mechanistic factors in development of T2D (12). Thus, protecting pancreatic β cell function is pivotal for T2D prevention (13). Both healthy diets and increased physical activity could help to preserve pancreatic β cells by increasing insulin sensitivity and improving the lipid profile thereby lowering the risk of T2D (14–16).

Nuts are now recognized as a healthy dietary component for reducing the risk of cardiovascular disease (17). The United States Department of Agriculture recommends consuming 5 servings [one-ounce eq (28.3 g) is considered as one serving] of nuts per week for adults on a 2,000 kcals/day diet (18). Almonds are low in carbohydrates and saturated fat but high in unsaturated fatty acids, especially MUFA. They are also a good source of plant protein, dietary fiber, phytosterols, polyphenols, vitamins, and minerals. Nuts consumption in general do not lead to weight gain in individuals with diabetes and also prevent T2D and metabolic syndrome (19–22). An earlier study among Asian Indian adults with diabetes showed that intake of 20% daily energy from almonds elicited protective effects on glycemic and CVD risk factors (23). However, additional studies are warranted in Indian adults who are overweight/obese, and for other NCDs. Hence, the present study was conducted to assess the effects of daily intake of 43 g (≈1.5 oz) of almonds for 12 weeks on insulin sensitivity, insulin resistance, and serum lipid markers among Asian Indian adults with overweight and obesity.

## Materials and methods

### Study participants

Participants for this parallel-arm, randomized, controlled trial were recruited based on pre-determined inclusion-exclusion criteria from the volunteers’ registry maintained at the Madras Diabetes Research Foundation, Chennai, India. In brief, the study included participants- aged 25–65 years (of both sexes), were overweight or obesity (BMI ≥ 23 kg/m^2^) (24). Participants with self-reported diabetes, history of allergy to nuts, using nutritional supplements, or on weight loss diets, pregnant/lactating women, and those with liver, kidney, thyroid, or other endocrine diseases and those using artificial dentures were excluded. The study was approved by the Institutional Ethics Committee at Madras Diabetes Research Foundation and was conducted in accordance with guidelines in the Declaration of Helsinki. The study was registered in the Clinical Trial Registry of India (CTRI 201710010251).

The methodology of this study is explained in detail elsewhere (25). All participants received a 1-week run in period with daily intake of 43 g (≈1.5 oz) of almonds followed by 2 weeks’ wash out period. An face to face interview schedule was used to assess participant compliance, i.e., willingness to consume almonds and also to check for adverse effects. After obtaining written informed consent, participants were randomized to either an intervention (*n* = 200) or control group (*n* = 200) using computer generated randomized numbers.

### Intervention

Participants in the intervention group received 43 g of raw almonds (≈1.5 oz) daily for 12 weeks. They were instructed to consume the almonds either as a mid-morning or evening snack, after adjusting for carbohydrate or fat calories in their regular diet under the guidance of a trained dietitian. The empty sachet of almonds was collected at the end of every month to monitor compliance with the intervention. Participants in the control arm adhered to their routine diet but were requested to avoid consuming nuts in any form during the 12-week study period. Further, all participants were advised not to make any major changes in lifestyle. The follow-up of participants every fortnight was conducted by trained research dietitians.

### Assessments

#### Anthropometric and clinical assessments

Body weight (kg) (electronic OMRON; Omron HBF 212, Tokyo, Japan), and waist circumference (cm) were measured using standard protocols (25). Body mass index (BMI) was calculated as weight (kg) divided by height in meter-squared (m^2^). The participants were requested to sit in upright position, relax for couple of minutes and blood pressure levels was measured in the left arm twice at an interval of 5 min using an electronic OMRON machine sitting in upright position (Omron corporation HEM 7120, Tokyo, Japan). The average of 2 readings for blood pressure and waist circumference was considered for analysis.

#### Biochemical assessments

Detailed methodology of assessments has been published elsewhere. In brief, after an overnight fast of at least 10 h, venous blood samples were obtained at baseline and at the end of week 12 by trained phlebotomists. Biochemical tests including: Oral glucose tolerance test after an 82.5 g oral glucose load (equivalent to 75 g of anhydrous glucose) was assessed with blood samples taken at 0 and 30 min for glucose and plasma insulin assessments, glycated hemoglobin (HbA1c), high sensitive -CRP, and lipid profile (serum cholesterol, LDL cholesterol, HDL cholesterol, triglycerides, apo A and apo B). Blood samples for C-peptide assessments were collected in fasting and post standard breakfast states (0 and 120 min) at baseline and at the end of week 12 as described earlier (25).

Plasma glucose was measured by the glucose oxidase peroxidase method; serum cholesterol by the cholesterol oxidase peroxidase-4-aminophenazone method; serum triglycerides by the glycerol phosphate oxidase-peroxidase-4-aminophenazone method; and HDL cholesterol directly with polyethylene glycol pre-treated enzymes using a Hitachi 912 Auto analyzer (Roche Diagnostics, GmbH, Mannheim, Germany) utilizing kits supplied by Boehringer Mannheim (Mannheim, Germany). LDL cholesterol was calculated using the Fried Wald formula in subjects with triglycerides ≤ 400 mg/dL. Hemoglobin A1c (HbA1c) was measured by high-performance liquid chromatography (HPLC) using a Variant machine (Bio-Rad, Hercules, CA, USA). Serum insulin concentration was assessed using a chemiluminescent immune assay method and insulin resistance by HOMA-IR using the formula:


$$\frac{\mathrm{HOMA}\,\mathrm{IR}=\mathrm{fasting}\,\mathrm{insulin}\,\left(\mathrm{microU}/L\right)}{\left(\mathrm{nmol}/L\right)/22.5}$$

Beta cell function was measured by the oral disposition index (DIo) which takes insulinogenic index and insulin sensitivity into account [DIo = (ΔI 0–30/ΔG0–30) X (1/fasting insulin)] (26).

#### Plasma fatty acid profiling

Lipids from plasma samples were as extracted in chloroform and methanol using a modified Folch method (27), converted to fatty acid methyl ester [FAME] (28) and were analyzed by gas chromatography (Shimadzu GC 2010 Plus with FID) equipped with Restek RT 2560 fused silica capillary column and a flame ionization detector. Nitrogen was used as a carrier gas, one microliter of sample was injected and the peaks were identified using FAME standards (37 fame mix, Supelco CRM 47885 Nu-Chek-Prep, Inc., Elysian, MN, USA). Percentages of plasma SFA, MUFA, and PUFA concentrations were calculated by summing the% concentrations of the respective FA within the treatment groups for each FA fraction.

### Diet and lifestyle

The baseline demographics, medical history, and lifestyle factors of participants were assessed using a screening questionnaire. A validated physical activity questionnaire (29) was completed at baseline and at the end of every month to collect information pertaining to the physical activity pattern of the participants. Two dietary 24-h recalls were administered monthly (1 weekday and 1 weekend) during the study as an additional measure of participants’ compliance.

### Continuous glucose monitoring (CGM) assessment

CGM was conducted in a subsample (*n* = 150) of the study population using an Abbott Freestyle Libre system. The CGM system consists of a sensor that records the interstitial fluid glucose levels every 15 min over a 24-h period for 14 consecutive days. The sensor was inserted in the back of the upper arm in participants who gave consent for CGM assessment. The interstitial glucose concentrations were assessed for 14 days at the beginning and at the end of week 12 as an additional measure of participant compliance. The CGM study was completed by 126 participants (58 in intervention and 68 in control group, respectively).

### Statistical analysis

Statistical analyses were performed using SAS 9.4 version (SAS Institute Inc., Cary, NC, USA). The data are reported as mean ± SD and n% for continuous and categorical variables, respectively. The within group changes were tested using independent *t*-tests for continuous variables and Pearson chi-square test for categorical variables. Between group differences for the outcome variables were tested using generalized linear model. The mean 24-h incremental Area Under the Curve for glucose (iAUC) (30) and Mean Amplitude of Glycemic Excursion (MAGE) were calculated to assess the glycemic variability using a validated algorithm (31). Significance was set at *p* < 0.05.

## Results

The study was completed by 352 out of 400 adults (178 and 174 participants in the control and intervention groups, respectively), 48 participants dropped out from the study due to various reasons including pregnancy, fear of blood sample collection, travel out of country and personal reasons and they were not considered for further analysis. The mean age and BMI of the study participants was 38 ± 9 years and 28.4 ± 3.8 kg/m^2^, respectively.

Table 1 shows the mean change in anthropometric, biochemical, and clinical characteristics of the participants between baseline and the end of week 12. There was a significant between group improvement in β cell function as assessed by insulin disposition index DIo [mean (95% CI) = 0.7 mmol/L (0.03, 1.5); *p* = 0.04] and reduction in insulin resistance assessed as HOMA-IR [−0.4 (−0.7, −0.04; *p* = 0.03] as well as total serum cholesterol [−5.4 mg/dl (−10.2, −0.6); *p* = 0.03] in the intervention group. There were also significant reductions in body weight, BMI, waist circumference, fasting glucose, and triglyceride levels in the intervention group relative to their baseline. The results were not adjusted for multiple comparisons as there is no consensus on whether or how this should be done.

**TABLE 1:** Mean change in anthropometric, biochemical and dietary characteristics of the study participants (*n* = 352).

Variables	Control group				Intervention group^$^				Between-group difference (95% CI)	p-value*
	Baseline (*n* = 178)	12 weeks (*n* = 178)	Change (*n* = 178)	*p*-value	Baseline (*n* = 174)	12 weeks (*n* = 174)	Change (*n* = 174)	*p*-value		
Body weight (kg)	71.8 ± 11.3	71.8 ± 11.5	0.0 ± 1.7	1.00	74.9 ± 10.4	74.5 ± 10.3	-0.3 ± 1.7	0.004	−0.4 (−0.7, 0.01)	**0.05**
Body mass index (kg/m^2^)	28.2 ± 3.8	28.1 ±3.8	0.0 ±0.7	0.78	28.6 ±3.7	28.5 ±3.6	-0.1 ±0.7	**0**.**04**	−0.1 (−0.3, 0.04)	0.14
Waist circumference (cm)	92.9 ±10.3	92.6 ±9.9	-0.3 ±3.4	0.23	94.7 ±9.6	93.9 ±9.1	-0.8 ±3.3	**0**.**001**	−0.5 (−1.3, 0.2)	0.14
Systolic blood pressure (mmHg)	115 ±14	114 ±14	-1.5 ±10.3	0.06	116 ±13	116 ±14	0.3 ±9.2	0.73	1.6 (−0.5, 3.7)	0.13
Diastolic blood pressure (mmHg) (mmHg)	78 ±10	78 ±10	-0.2 ±8.3	0.81	79 ±10	79 ±10	-0.1 ±7.3	0.80	0.1 (−1.6, 1.8)	0.89
Fasting blood glucose (mg/dl)	94 ±12	94 ±12	-0.5 ±10.5	0.56	95 ±11	93 ±10	-2.0 ±8.6	**0**.**002**	−1.9 (−3.9, 0.1)	0.07
HOMA IR	3.3 ±1.7	3.5 ±2.0	0.2 ±1.6	0.08	3.3 ±1.7	3.3 ±1.7	-0.1 ±1.5	0.86	−0.4 (−0.7, -0.04)	**0.03**
Oral disposition index DIo (mmol/l)	3.2 ±4.9	3.3 ±4.3	0.2 ±4.1	0.55	2.7 ±3.3	3.7 ±3.2	0.9 ±3.5	0.28	0.7 (0.03, 1.5)	**0.04**
HbA1c (%)	6 ±0.5	6 ±1	0 ±0.1	0.24	6 ±0.5	6 ±0.5	0 ±0.1	0.17	0 (-0.03, 0.03)	0.97
C Peptide (0 min) pmol/ml	1 ±0.4	1 ±0.4	0.0 ±0.3	0.20	1 ±0.3	1 ±0.4	0 ±0.3	0.51	0 (−0.1, 0.1)	1.00
C Peptide (120 min) pmol/ml	2 ±1	2 ±1	-0.2 ±0.8	**0**.**02**	2 ±1	2 ±1	-0.1 ±0.9	0.09	0.1 (−0.1, 0.3)	0.29
Total serum cholesterol (mg/dl)	191 ±32	194 ±35	3 ±21	0.05	194 ±40	192 ±39	-2.4 ±23.7	0.18	−5.4 (−10.2, −0.6)	**0.03**
^#^Serum triglyceride (mg/dl)	123 ±63	123 ±76	-0.5 ±45.0	0.89	134 ±70	127 ±64	-7.7 ±40.9	**0**.**01**	−6.8 (−16.0, 2.3)	0.14
Serum low density lipoprotein (mg/dl)	126 ±29	130 ±32	3.5 ±19.1	**0**.**02**	128 ±33	128 ±34	-0.5 ±21.7	0.74	−4 (−8.5, 0.4)	0.07
Serum high density lipoprotein (mg/dl)	41 ±8.0	41 ±7.9	-0.4 ±5.1	0.34	40 ±8	39 ±8	-0.3 ±4.8	0.35	0.1 (−1.0, 1.2)	0.87
Apo A lipoprotein (mg/dl)	122 ±25	126 ±21	4.2 ±31.1	0.07	126 ±23	126 ±23	-0.2 ±30.9	0.92	−3.4 (−10.1, 3.2)	0.31
Apo B lipoprotein (mg/dl)	84 ±55	82 ±22	-2 ±58	0.60	82 ±23	82 ±23	-0.1 ±28.8	0.95	3.4 (−6.4, 13.1)	0.50

Data presented as mean ± SD; ^#^Log transformed.

^$^Raw almond 43 g/d (Intervention) for 12 weeks.

*p-value < 0.05 considered as significant using generalized linear model.

The values in bold shows the variables that are significantly different.

The mean change in nutrient profile of the study participants assessed using average of 24-h dietary recalls collected during the study period shown in Table 2 elicited a significant decrease in carbohydrate (% energy as well as g/d) by almost 13%; (*p* < 0.001) and concurrent increase in intake of energy from fat (% energy as well as g/d) (0.7 ± 4.7 vs. 8.6 ± 5.7; *p* < 0.001), especially MUFA and PUFA in the intervention group compared to control. The intake of dietary fiber (g/d) and protein (g/d) also significantly increased in the intervention group [2.3 (0.7, 3.9); *p* = 0.004; 3.1 (0.3, 5.8); *p* = 0.03].

**TABLE 2:** Mean change in dietary characteristics of the study participants (*n* = 352).

Variables	Control group				Intervention group^$^				Between-group difference (95% CI)	*p*-Value*
	Baseline (*n* = 178)	12 weeks (*n* = 178)	Change (*n* = 178)	*p*-value	Baseline (*n* = 174)	12 weeks (*n* = 174)	Change (*n* = 174)	*p*-value		
Energy (Kcal)	1551 ± 447	1549 ± 351	-2 ± 377	0.94	1598 ± 464	1683 ± 360	84 ± 359*	**0**.**002**	86.3 (9.6, 162.9)	**0**.**03**
Carbohydrates (%E)	61.5 ± 5.8	60.6 ± 6.2	-0.9 ± 5.9	0.06	60.5 ± 5.5	51.7 ± 5.0	-8.8 ± 6.1*	**<0**.**0001**	−8.0 (−9.2, −6.7)	**<0**.**0001**
Carbohydrates (g)	236 ± 66	231 ± 48	-3.7 ± 56	0.38	239 ± 66	217 ± 52	-22 ± 55	**<0**.**001**	−18.6 (−30.2, −7.1)	**0**.**002**
Protein (%E)	12.0 ± 1.6	12.2 ± 1.3	0.2 ± 1.8	0.07	12.1 ± 1.5	12.4 ± 1.2	0.3 ± 1.7*	**0**.**01**	0.1 (−0.3, 0.5)	0.61
Protein (g)	47 ± 15	48 ± 13	1.1 ± 13	0.26	48 ± 16	53 ± 13	4.1 ± 14	**<0**.**001**	3.1 (0.3,5.8)	0.03
Dietary fiber (g)	24.0 ± 8.5	22.8 ± 5.6	-1.2 ± 7.6	**0**.**04**	25.2 ± 8.0	26.3 ± 6.2	1.1 ± 7.4	0.05	2.3 (0.7, 3.9)	**0**.**004**
Total fat (%E)	25.2 ± 5.5	25.9 ± 4.6	0.7 ± 4.7	0.05	26.0 ± 4.9	34.6 ± 4.7	8.6 ± 5.7*	**<0**.**0001**	7.9 (6.8, 9.0)	**<0**.**0001**
Total fat (g)	44 ± 18	45 ± 14.9	0.7 ± 14.9	0.51	48 ± 19	65 ± 16	18 ± 16	**<0**.**0011**	16.8 (13.5,20.0)	**<0**.**001**
SFA (%E)	8.3 ± 3.4	8.6 ± 2.7	0.3 ± 3.6	0.25	8.1 ± 3.1	8.4 ± 2.2	0.3 ± 3.3	0.22	0.0 (−0.7, 0.7)	0.98
MUFA (%E)	6.4 ± 1.7	6.7 ± 1.4	0.3 ± 1.8	0.05	6.5 ± 1.5	12.8 ± 2.8	6.3 ± 3.0*	**<0**.**0001**	6.0 (5.5, 6.5)	**<0**.**0001**
PUFA (%E)	9.0 ± 2.2	9.4 ± 1.9	0.4 ± 2.6	0.06	9.4 ± 2.1	11.1 ± 1.7	1.7 ± 2.3*	**<0**.**0001**	1.3 (0.8, 1.8)	**<0**.**0001**
Linoleic acid (%E)	8.6 ± 2.3	8.9 ± 2.0	0.3 ± 2.4	0.07	8.9 ± 2.2	10.6 ± 2.0	1.7 ± 2.5*	**<0**.**0001**	1.4 (0.8, 1.9)	**<0**.**0001**
Linolenic acid (%E)	0.2 ± 0.1	0.2 ± 0.1	0.0 ± 0.1	0.91	0.2 ± 0.1	0.2 ± 0.2	0.0 ± 0.2	0.20	0.0 (−0.1, 0.0)	0.26

Data presented as mean ± SD; ^$^Raw almond 43 g/d (Intervention) for 12 weeks.

*p-Value < 0.05 considered as significant using generalized linear model.

Baseline: Average of 3 recalls; MUFA, monounsaturated fatty acids; PUFA, polyunsaturated fatty acids; SFA, saturated fatty acids; MUFA, monounsaturated fatty acids; PUFA, polyunsaturated fatty acids; SFA, saturated fatty acids.

The values in bold shows the variables that are significantly different.

**FIGURE 1:**
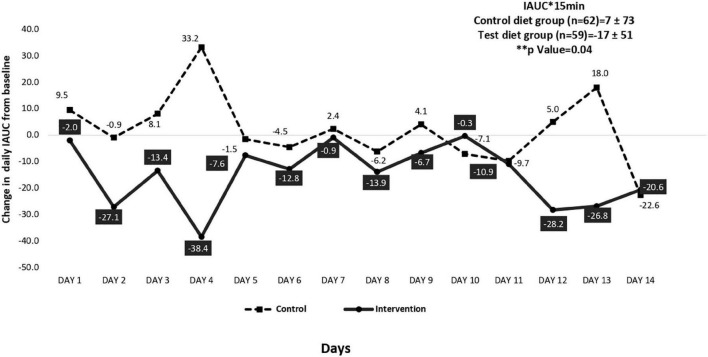
Mean change in incremental area under the curve (IAUV-mg/dl) assessed by continuous glucose monitoring (CGM) system for control and intervention group participants from baseline (*n* = 121).

**FIGURE 2:**
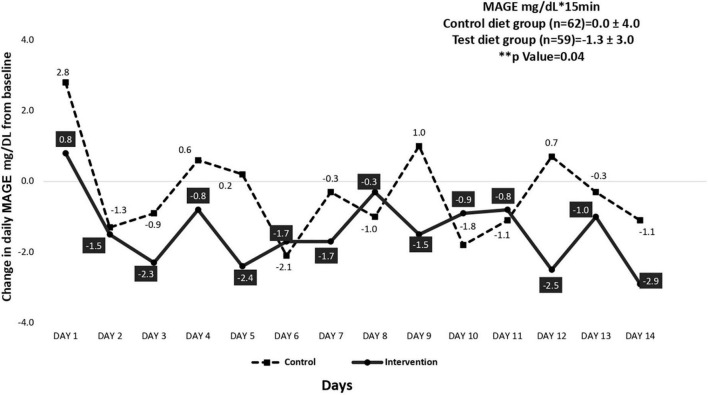
Mean change in mean amplitude glycemic excursions (MAGE) assessed by continuous glucose monitoring (CGM) system for control and intervention group participants from baseline (*n* = 121).

Figures 1, 2 illustrate the mean change in IAUC and MAGE of the intervention and control group participants. At baseline, CGM assessments were carried out in a sub sample of 150 participants (intervention, *n* = 72 and control, *n* = 78). However, only 127 participants (intervention, *n* = 59 and control, *n* = 68) completed the assessment. There was a significant 12% reduction (*p* = 0.03) in IAUC from baseline in the intervention group compared to the control group (Figure 1). Glycemic variability measured as MAGE also showed a significant 10% reduction (from baseline) in the intervention group compared to control group (*p* = 0.04) (Figure 2).

The mean change in plasma fatty acids of participants assessed as a measure of compliance with the intervention is presented in Supplement Table 1. The MUFA% (mean ± SD: 40.3 ± 13.9 vs. 43.0 ± 14.3) and PUFA% (25.2 ± 12.6 vs. 27.3 ± 12.7) content significantly increased in the intervention group compared to control group thereby supporting their compliance with the almond’s intervention. Myristoleic and palmitoleic in with respect to MUFA, linoleic acid and total n6 in PUFA significantly increased in the intervention group.

## Discussion

In this randomized, controlled trial conducted over-weight Asian Indian adults the consumption of 43 g (≈1.5 oz) of almonds daily as a snack (mid -morning/evening) over 12 weeks induced decreases in total body weight, BMI, and waist circumference. There was an increase in insulin secretion as assessed by oral disposition index, a reduction in insulin resistance (HOMA-IR), and serum total cholesterol levels relative to the control group participants. Almonds also improved the intake of dietary fiber and healthy fats particularly MUFA and helped to reduce the carbohydrate content of the diet.

Many studies have assessed the effect of almonds on glycemic and insulin markers, indicating that almond-enriched diets significantly improve β cell function (32–34). In the present study, intake of almonds significantly improved oral disposition index (DIo) which is considered a reliable index of β cell function (35, 36). An earlier study among adults with prediabetes consuming 60 g of almonds per day (20% energy) over 16 weeks showed a significant reduction in HOMA-IR compared to the control group (34) which is comparable to our study findings. Studies have shown that nuts may decrease glycemic excursions and enhance insulin secretion when consumed with high carbohydrate foods (37). Lu et al. (38) have shown that a nut-based snack lowers postprandial glycemic response in high-risk, overweight, pre-diabetic adults. The present study with almonds consumed as a snack, showed a similar reduction in glycemic excursions measured as incremental area under the curve (IAUC) and as mean amplitude glycemic excursion (MAGE) compared to the control group. This may potentially reduce diabetes risk at the long term. A pooled meta-analysis concluded that almond consumption improved the glycemic profile of both healthy individuals as well as those with pre-diabetes despite its high fat content (39). However, there are, some studies that report almond intake has no effect on glycemic parameters (40, 41).

Jenkins et al. (41) reported a reduction in 24-h urinary C-peptide output after consumptions of muffins containing half (37 ± 2 g/d) and full dose (73 ± 3 g/d) almonds compared to the control group (muffins without almonds), but in our study, no changes in serum C- peptide (fasting and stimulated) were observed. Evidence suggests that the fatty acid composition of almonds could be cardio protective through inducing a decrease in cholesterol levels, in the present study, serum total cholesterol significantly decreased in the intervention group. Previous studies with almonds have shown improvement of blood lipid profiles including total cholesterol, triglyceride, LDL and HDL in participants with obesity, hyperlipidemia and diabetes (42–44). In a study in Indian adolescents and young adults, almonds supplemented in the diet reduced total as well as LDL-C cholesterol (21). Studies among Asian Indian adults with metabolic syndrome and T2D have also shown significant reductions in total and LDL cholesterol levels (23, 45). A randomized control trial supplementing almonds (10 g/d) in patients with coronary artery disease observed significant reductions in total, LDL and VLDL cholesterol and LDL:HDL cholesterol ratios (*p* < 0.05) in addition to a significant improvement in HDL cholesterol (46). However, in the present study HDL cholesterol was unchanged as shown by an earlier Indian study (23). Similar findings were also reported by Liu et al. (47) and Palacios et al. (40) and also meta-analysis of randomized controlled trials by Phung et al. (48) including 25–168 g/d almonds did not reveal statistically significant effects on HDL (48).

In a study among adults with hyperlipidemia, Nishi et al. (49) showed that almond consumption favorably alters the serum fatty acid profile by increasing the proportions of total MUFA (oleic acid in particular) and decreasing saturated fatty acids. These findings correlate with improvements in blood lipoproteins and with a decreased 10-year coronary heart disease risk. In another study among adults with obesity, plasma polyunsaturated fatty acids (eicosapentaenoic and docosahexaenoic acids in particular) significantly increased post-intervention with 15 g each of walnuts and almonds for 8 weeks (50). Similarly, to the present study, plasma MUFA-oleic acid and PUFA especially *n6* significantly increased in the intervention group compared to the control group. There was a significant increase in eicosapentaenoic and docosahexaenoic acids within the intervention group.

Because of their high fat content, nuts are often avoided because of fear of weight gain. On the contrary, nuts are proven to be satiating and do not promote weight gain (39). The intake of nuts has in fact been inversely associated with the prevalence of central obesity and metabolic syndrome (51). Some studies among healthy individuals and those with hyperlipidemia have reported that 68 and 73 g of almonds, respectively, have no effect on weight (52, 53). Several other studies have also observed no change in body weight post almond intake (34, 52–54). Abazarafad et al. (42) in a randomized controlled trial showed that 50 g of almonds taken over a period of 3 months prompted significant reductions in BMI, waist circumference and body weight from baseline compared to a “nut-free group.” Several mechanisms have been proposed to explain decrease in body weight with nuts. Recent, meta regression by Nishi et al. (55) showed contrary to the myth-higher nut intake was associated with reduction in body weight and body fat, hence the fear of eating nuts and body weight gain is perhaps redundant.

By study design, changes were made in the dietary profile of the study participants wherein there was a significant reduction in carbohydrate and increase in intake of dietary fiber and energy from fat in the intervention group. In another study among participants with prediabetes (40) daily intake of 3 oz (85 g) of almonds not only lowered the carbohydrate intake but also elicited an increase in intake of protein, dietary fiber and unsaturated fats. This is similar to the findings reported in the present study. Hence, the Asian Indian diets which are high in carbohydrate and low in MUFA and dietary fiber may benefit with the inclusion of nuts like almonds to improve the overall diet quality.

The mechanism by which almonds favorably influence the glycemic, insulin, and lipid markers is not clear. Increasing insulin sensitivity reduces insulin secretion, which, in turn, reduces the load on the β-cell and thereby helps in the prevention or delay of pancreatic β-cell dysfunction (40). Almonds are low in carbohydrate and saturated fat while rich in unsaturated fatty acids, especially MUFA - Oleic acid which are readily oxidizable and increase the thermogenic effect of food compared to saturated fatty acids. Gadgil et al. (56) showed that partial replacement of carbohydrates with unsaturated fatty acids (Mediterranean diet) could improve insulin sensitivity in populations at risk of CVD. Almonds are also a vital source of plant protein, dietary fiber, several phytosterols, polyphenols, vitamins, and minerals. These bioactive compounds may help to regulate body weight, improve metabolic syndrome and thereby improve insulin sensitivity and reduce resistance. The bioactive phytosterols in almonds compete with dietary cholesterol and bile acids for uptake in mixed micelles, thus interfering with cholesterol and bile acid absorption. Another possible mechanism of action of nuts favoring better glycemic and insulinemic responses could be due to reduced gastric emptying rate due to the presence of higher fat and protein (57). This also helps to increase satiety thereby reducing additional intake of food. Incomplete mastication of nuts lead to loss of energy in the stool which may aid in weight management (58). The plant protein and L-arginine in almonds have been shown to have cholesterol-lowering effects, possibly by altering macronutrient metabolism and disrupting enterohepatic homeostatic regulation, respectively (56, 57, 59). Thus, the mechanism of action for almonds on glycemic, insulin, and cholesterol are mainly regulated by its nutrient composition and bioactive components and may be multifactorial.

The strengths of the present study are the large sample size, good response rate, and standardized anthropometric assessments and laboratory measurements. Further, data collection was carried out *via* face-to-face interview by well-trained research dietitians. Dietary compliance was measured both by nutrient biomarker (plasma fatty acid) and self-reported dietary intake of MUFAs and fiber suggesting good adherence. One of the limitations is that the study is restricted to Asian Indians with overweight and obesity and therefore the results may not be generalizable to other populations. Because this was a free-living trial, there is a possibility that peer, family, and environmental constraints impacted the study findings of both the groups, biasing results toward the null. The 24-h dietary recalls collected during the study to assess participants’ adherence may have suffered from recall bias or may not have reflected typical intake, but these are inherent biases in such feeding trials.

## Conclusion

This study conducted on Asian Indian adults with overweight and obesity indicates that consuming 43 g of almonds daily as a snack over a period of 12 weeks results in improvement in insulin resistance and β cell function in addition to lowering of serum total cholesterol and body weight. Plasma fatty acid measurements confirmed a significant increase in plasma oleic acid in the almond group suggesting that almonds helped to improve the dietary intake of MUFA. These findings support a role for almonds as a healthy snack alternative or incorporated into meals for Asian Indians who are predisposed to T2D and CVD. These effects from almonds in the long run could aid in reducing the risk of diabetes and other cardiometabolic diseases in Asian Indians.

## Data availability statement

The raw data supporting the conclusions of this article will be made available by the authors, without undue reservation.

## Ethics statement

The studies involving human participants were reviewed and approved by the Institutions Ethics Committee at Madras Diabetes Research Foundation. The patients/participants provided their written informed consent to participate in this study.

## Author contributions

VM, RA, and VS conceived the study. KK, JS-S, WW, and RM reviewed the protocol of the study. RG, KA, NK, and VK executed the trial. SS, RJ, and KP assessed plasma biomarkers. RG and KA performed the statistical analysis. VM, WW, RM, JS-S, KK, RA, RU, and VS further assisted in interpretation. RG initiated the manuscript and VS, NK, and VSM further assisted. VM, WW, RM, JS-S, KK, RA, RU, KG, DB, RP, and VS critically reviewed the manuscript and approved the final version. All authors contributed to the article and approved the submitted version.
