# Supplementary material for: Effect of almond consumption on insulin sensitivity and serum lipids among Asian Indian adults with overweight and obesity– A randomized controlled trial
Source: Front Nutr. 2023 Jan 10;9:1055923. doi: 10.3389/fnut.2022.1055923 (PMC9873375; doi:10.3389/fnut.2022.1055923)
Supplement: Supplementary file 1 [file Table_1.DOCX]

**Supplement table 1: Mean change in percent plasma fatty acid profile of the study participants (n=352)**

| **Carbon Chain length** | **Fatty acids** | **Control (n=178)** | | | **Intervention (n=174)** | | | **Between groups p value** |
| --- | --- | --- | --- | --- | --- | --- | --- | --- |
|  |  | **Baseline** | **End** | **p value** | **Baseline** | **End** | **p value** |  |
| **C12:0** | Lauric acid | 1.0 ± 3.0 | 0.7 ± 1.6 | 0.10 | 0.6 ± 1.5 | 0.7 ± 1.9 | 0.48 | 0.11 |
| **C13:0** | Tridecanoic acid | 7.0 ± 6.0 | 7.3 ± 6.2 | 0.21 | 5.9 ± 5.5 | 4.2 ± 5.3 | <.0001 | 0.18 |
| **C14:0** | Myristic acid | 0.1 ± 0.6 | 0.1 ± 0.4 | 0.60 | 0.1 ± 0.5 | 0.1 ± 0.5 | 0.20 | 0.24 |
| **C15:0** | Pentadecanoic acid | 8.8 ± 8.3 | 8.9 ± 8.5 | 0.74 | 9.6 ± 9.0 | 8.8 ± 8.0 | 0.11 | 0.32 |
| **C16:0** | Palmitic acid | 3.3 ± 7.4 | 3.2 ± 7.4 | 0.82 | 4.0 ± 8.2 | 5.0 ± 8.5 | 0.02 | 0.04 |
| C17:0 | Heptadecanoic acid | 4.6 ± 4.1 | 4.3 ± 4.0 | 0.19 | 4.1 ± 4.2 | 3.8 ± 4.1 | 0.20 | 0.54 |
| **C18:0** | Stearic acid | 2.4 ± 4.7 | 2.8 ± 4.7 | 0.06 | 1.9 ± 3.4 | 1.1 ± 2.9 | <.0001 | 0.87 |
| **C20:0** | Arachidic acid | 1.6 ± 3.6 | 1.4 ± 2.6 | 0.35 | 1.8 ± 3.7 | 2.7 ± 5.3 | <.0001 | 0.002 |
| **C21:0** | Heneicosanoic acid | 0.9 ± 1.6 | 0.7 ± 1.0 | 0.10 | 0.6 ± 1.8 | 0.7 ± 2.0 | 0.03 | 0.09 |
| **C22:0** | Behenic acid | 0.3 ± 1.4 | 0.3 ± 1.3 | 0.92 | 0.3 ± 1.2 | 0.2 ± 0.8 | 0.26 | 0.23 |
| **C23:0** | Tricosanoic acid | 4.0 ± 4.6 | 3.6 ± 4.4 | 0.17 | 3.7 ± 4.9 | 4.1 ± 4.6 | 0.09 | 0.92 |
| **C24:0** | Lignoseric acid | 0.2 ± 0.6 | 0.3 ± 0.6 | 0.41 | 0.3 ± 0.8 | 0.4 ± 0.8 | 0.18 | 0.31 |
|  | **SUM Saturated fatty acids** | 34.3 ± 21.2 | 33.5 ± 20.0 | 0.09 | 32.7 ± 22.0 | 31.9 ± 21.6 | 0.06 | 0.86 |
| **C14:1** | Myristoleic acid | 0.4 ± 0.9 | 0.4 ± 0.8 | 0.69 | 0.4 ± 0.7 | 0.6 ± 1.2 | **0.002** | **<.0001** |
| **C15:1** | Pentadecanoic acid | 3.0 ± 5.0 | 3.2 ± 5.3 | 0.27 | 3.1 ± 4.9 | 3.5 ± 5.2 | **0.02** | 0.76 |
| **C16:1** | Palmitoleic acid | 15.7 ± 12.9 | 15.8 ± 12.5 | 0.88 | 13.5 ± 12.5 | 14.6 ± 13.1 | **0.0001** | **<.0001** |
| **C17:1** | Heptadecenoic acid | 1.6 ± 3.2 | 1.4 ± 2.5 | 0.14 | 1.5 ± 2.6 | 1.5 ± 2.6 | 0.69 | 0.22 |
| **C18:1n9t** | Elaidic acid | 9.0 ± 8.0 | 8.7 ± 9.0 | 0.60 | 8.2 ± 8.0 | 8.3 ± 7.8 | 0.74 | 0.65 |
| **C18:1n9c** | Oleic acid | 8.0 ± 12.9 | 7.8 ± 11.7 | 0.86 | 11.1 ± 14.2 | 11.7 ± 14.0 | 0.17 | 0.87 |
| **C18:1n9** | Elaidic +oleic | 17.0 ± 12.5 | 16.5 ± 11.2 | 0.27 | 19.3 ± 12.6 | 20.1 ± 12.5 | **0.04** | 0.05 |
| **C20:1** | Cis-11-eicosenoic acid | 1.4 ± 5.8 | 1.6 ± 5.8 | 0.07 | 1.2 ± 5.5 | 1.3 ± 6.0 | 0.20 | 0.31 |
| **C22:1n9** | Erucic acid | 1.3 ± 1.4 | 1.4 ± 1.4 | 0.49 | 1.3 ± 1.5 | 1.3 ± 1.5 | 0.77 | 0.51 |
| **C24:1** | Nervonic acid | 0.1 ± 0.4 | 0.2 ± 1.0 | 0.28 | 0.1 ± 0.3 | 0.1 ± 0.2 | 0.42 | 0.71 |
|  | **SUM Mono unsaturated fatty acids** | 40.6 ± 14.6 | 40.5 ± 14.1 | 0.87 | 40.3 ± 13.9 | 43.0 ± 14.3 | **<.0001** | **<.0001** |
| **C18:2n6t** | Linolelaidic acid | 1.3 ± 4.6 | 1.4 ± 4.9 | 0.47 | 0.9 ± 4.1 | 0.7 ± 2.9 | 0.22 | 0.20 |
| **C18:2n6c** | Linoleic acid acid | 10.5 ± 7.5 | 10.4 ± 7.4 | 0.63 | 11.1 ± 7.8 | 12.3 ± 9.3 | **0.02** | **<.0001** |
| **C18:2n6** | Linolelaidic+ linoleic acids | 11.8 ± 7.4 | 11.8 ± 7.3 | 0.85 | 12.0 ± 7.8 | 12.9 ± 9.3 | **0.04** | **0.0003** |
| **C18:3n6** | Gamma-linolenic acid | 6.8 ± 8.2 | 6.4 ± 7.9 | 0.13 | 6.2 ± 7.7 | 6.1 ± 7.8 | 0.68 | 0.61 |
| **C18:3n3** | Alpha Linolenic acid | 0.2 ± 0.6 | 0.3 ± 0.7 | 0.42 | 0.2 ± 0.6 | 0.2 ± 0.9 | 0.97 | 0.69 |
| **C20:2** | Eicosadienoic acid | 0.4 ± 1.0 | 0.4 ± 1.0 | 0.21 | 0.4 ± 0.9 | 0.5 ± 1.1 | 0.12 | 0.46 |
| **C20:3n6** | Dihomo-gamma-linolenic acid | 1.1 ± 1.5 | 1.3 ± 1.5 | 0.23 | 1.2 ± 1.7 | 1.3 ± 1.7 | 0.15 | **0.01** |
| **C20:3n3** | Eicosatrienoic acid | 0.7 ± 1.3 | 0.7 ± 1.6 | 1.00 | 0.8 ± 1.6 | 0.7 ± 1.6 | **0.05** | 0.84 |
| **C20:4n6** | Arachidonic acid | 1.9 ± 4.4 | 2.0 ± 4.7 | 0.30 | 2.1 ± 4.7 | 2.5 ± 5.3 | **0.05** | 0.53 |
| **C20:5n3** | Eicosapentaenoic acid | 0.2 ± 0.5 | 0.3 ± 1.6 | 0.17 | 0.1 ± 0.4 | 0.2 ± 0.6 | **0.05** | 0.18 |
| **C22:2** | Docosadienoic acid | 0.0 ± 0.1 | 0.0 ± 0.3 | 0.69 | 0.0 ± 0.2 | 0.1 ± 0.3 | 0.07 | 0.31 |
| **C22:6** | Docosahexaenoic acid | 2.1 ± 7.3 | 1.9 ± 7.1 | 0.32 | 2.0 ± 5.7 | 2.7 ± 6.4 | **0.01** | 0.34 |
|  | Total n-6 PUFA | 22.2 ± 9.5 | 21.9 ± 9.7 | 0.27 | 22 ± 9.8 | 23.3 ± 10.5 | **0.001** | **0.001** |
|  | Total n-3 PUFA | 10.3 ± 10.2 | 9.9 ± 10 | 0.36 | 9.6 ± 9.1 | 10.4 ± 9.3 | **0.05** | 0.09 |
|  | **SUM Poly unsaturated fatty acids** | 25.3 ± 11.8 | 25.1 ± 12.1 | 0.29 | 25.2 ± 12.6 | 27.2 ± 12.7 | **<.0001** | **<.0001** |
